# Dataset describing the development, optimization and application of SRM/MRM based targeted proteomics strategy for quantification of potential biomarkers of EGFR TKI sensitivity

**DOI:** 10.1016/j.dib.2018.04.086

**Published:** 2018-05-02

**Authors:** Shivangi Awasthi, Tapan Maity, Benjamin L. Oyler, Xu Zhang, David R. Goodlett, Udayan Guha

**Affiliations:** a Thoracic & Gastrointestinal Oncology Branch, Center for Cancer Research, NCI, Bethesda, MD, United States; b School of Medicine, University of Maryland, Baltimore, MD, United States; c School of Pharmacy, University of Maryland, Baltimore, MD, United States

## Abstract

The data presented here describes the use of targeted proteomic assays to quantify potential biomarkers of Epidermal growth factor receptor (EGFR) tyrosine kinase inhibitor (TKI) sensitivity in lung adenocarcinoma and is related to the research article: “Quantitative targeted proteomic analysis of potential markers of tyrosine kinase inhibitor (TKI) sensitivity in EGFR mutated lung adenocarcinoma” [1]. This article describes the data associated with liquid chromatography coupled to multiple reaction monitoring (LC-MRM) method development which includes selection of an optimal transition list, retention time prediction and building of reverse calibration curves. Sample preparation and optimization which includes phosphotyrosine peptide enrichment via a combination of pan-phosphotyrosine antibodies is described. The dataset also consists of figures, tables and Excel files describing the quantitative results of testing these optimized methods in two lung adenocarcinoma cell lines with EGFR mutations.

**Specifications table**

**Table:** 

Subject area	*Clinical Chemistry, Biology*
More specific subject area	*Targeted therapy response biomarkers in lung adenocarcinoma*
Type of data	*Tables, graphs, figures, Excel file*
How data was acquired	*MS data acquired with LTQ-Orbitrap Elite (Thermo Scientific) and Agilent Triple Quadrupole 6495*
Data format	*Analyzed and filtered (figures and tables)*
Experimental factors	*Immunoprecipitation for enrichment of tyrosine phosphorylated peptides. Synthetic standard phosphorylated peptides spiked in for quantification.*
Experimental features	*Spectral library generation and retention time prediction for scheduled chromatography, optimization of enrichment strategy for phosphotyrsoine target peptides and external multi-point reverse response curve generation for quantitation*
Data source location	*Bethesda, MD, USA*
Data accessibility	*All the datasets are provided within this article and as a supplementary Excel file.*

**Value of the data**

• The dataset describes the optimization and method development for building quantitative targeted proteomic assays for phosphotyrosine peptides.
• The methods and data presented here can be used for building similar MRM assays for phosphopeptide quantification and verification of quantitative phosphorylation results observed in large-scale LC-MS based phosphoproteomic experiments.
• The data describing the approach of using the heavy labelled synthetic standards and immunoaffinity enrichment of the tyrosine phosphorylated peptides can be applied to interrogate these targets in other cell-based models and tumor tissue from patients.

## 1 Data

The data presented here describe the development of LC-MRM based methods for quantification of tyrosine phosphorylated peptide biomarkers in lung adenocarcinoma cells. The experimental design consisted of development of robust MRM methods for each phosphorylated peptide candidate using synthetic phosphorylated peptides as “spike-in” standards. These assays were implemented in lung adenocarcinoma cells harboring TKI-sensitive EGFR^L858R^ (H3255) and -resistant EGFR^L858R/T790M^ (H1975) mutants, with and without 1st generation TKI, erlotinib and 3rd generation TKI, osimertinib treatment in 3–6 biological replicates.

## 2 Experimental design, materials and methods

### 2.1 Spectral library generation and retention time approximation

Previously published LC-MS output files [2] based on data-dependent acquisition (DDA) were used to generate a spectral library in Skyline. Briefly, enriched phosphopeptide samples were analyzed on a LTQ-Orbitrap Elite (Thermo Scientific Corp., San Jose, CA) coupled to an Easy-nLC 1000 system (Thermo Scientific Corp., San Jose, CA). Peptides were trapped on a 100 µm i.d. × 2 cm long precolumn (Acclaim PepMap100 Nano Trap column, C18, 5 µm, 100 Å). Subsequent peptide separation was carried out on a nano-LC column (Acclaim PepMap100, C18, 3 µm, 100 Å, 75 µm i.d. × 25 cm, nanoViper). Mobile phase A consisted of 0.1% formic acid in water (v/v) and mobile phase B consisted of 0.1% formic acid in 90% acetonitrile. For each liquid chromatography-tandem mass spectrometry (LC-MS/MS) analysis, peptides were eluted from the column at 250 nL/min using an acetonitrile gradient of 2–8% B in 8 min, 8–32% B over 100 min, 32–100% B in 10 min and held at 100% B for an additional 10 min. The eluting peptides were interrogated with an Orbitrap analyzer with full scan spectra acquired between *m/z* 350 and 1800 at resolution of 120,000 followed by data-dependent HCD MS/MS acquisition for the top 10 most abundant ions at 32% normalized collision energy.

**Fig. 1:**
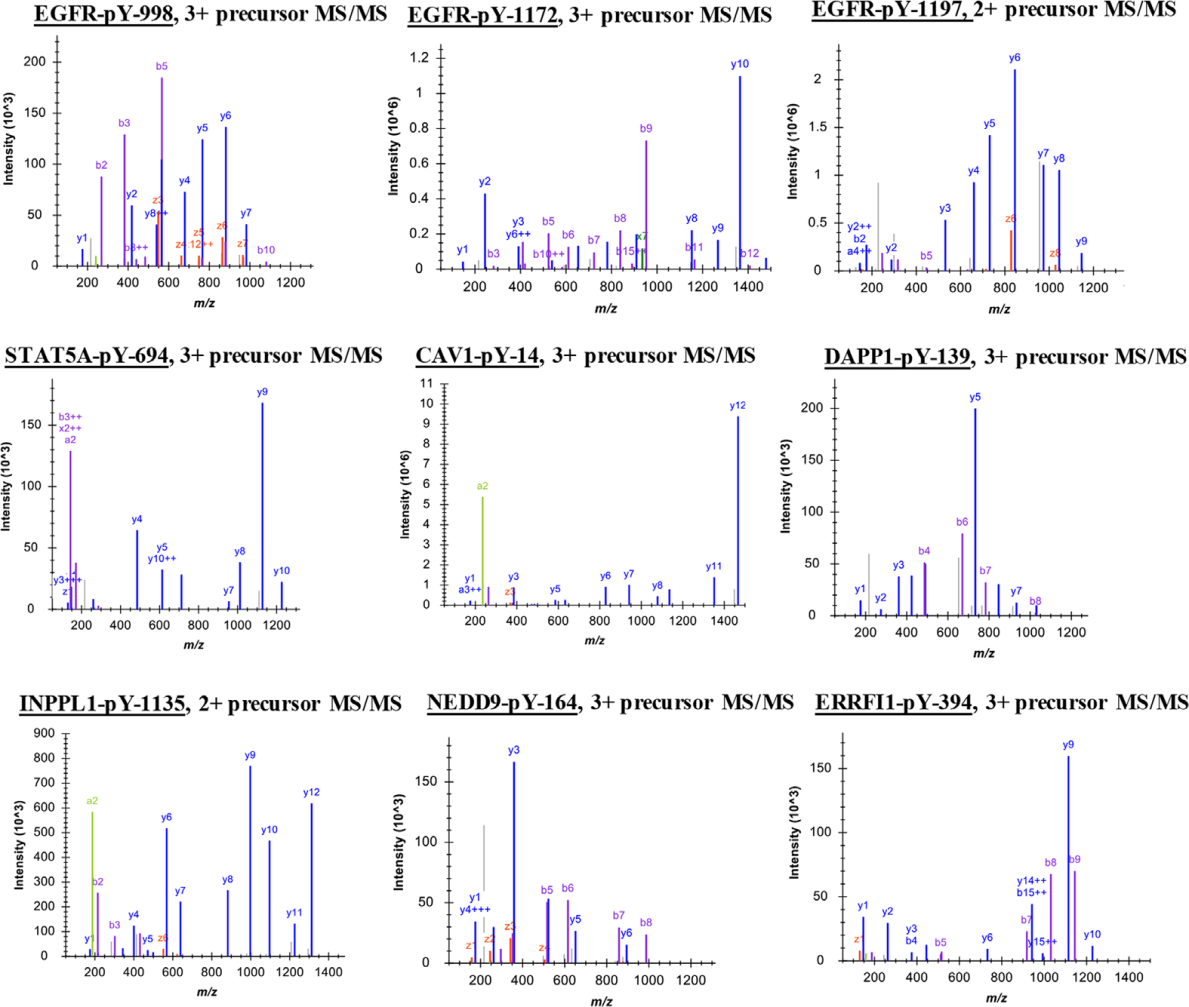
Spectral library built from previously performed Data Dependent Acquisition (DDA) experiments to facilitate selection of optimal transitions.

The resulting raw files were searched against the Uniprot human protein database using the Maxquant software (version 1.3.0.5) with Andromeda search engine using previously described parameters [2]. The resulting search output file *msms.txt* was uploaded in Skyline to build the spectral library to pick optimal transitions for the construction of the LC-MRM transition list. The annotated MS/MS spectra for 9 of the 11-selected tyrosine phosphorylated peptide targets are shown in Fig. 1. For the remaining three peptides containing DAPP1-pY139, AHNAK-pY715 and -pY160 phosphosites, individual injections of the heavy labelled peptide standard carried out on the nano-chip-LC using a 1260 Infinity Series HPLC-Chip cube interface (Agilent, Palo Alto, CA) coupled to a 6495-triple quadrupole mass spectrometer (Agilent, Palo Alto, CA) identified a different charged precursor ion that was more abundant compared to the spectra obtained from HCD MS/MS in the OrbitrapElite. Hence, for these three phosphopeptides, MRM data was used for the selection of optimal transitions.

**Fig. 2:**
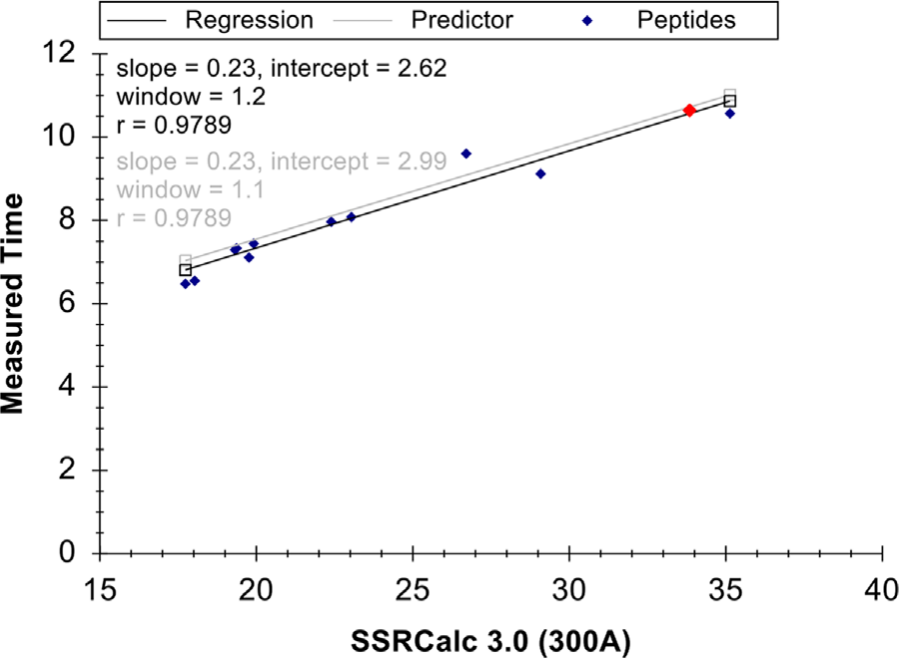
The linear regression obtained for retention time prediction using the SSRCalc 3.0 calculator.

Using the list of optimal transitions (Table 1), an unscheduled MRM method with a dwell time of 50 ms and a cycle time of 700 ms was used to determine the retention times of the targets and to generate scheduled MRM methods. The correlation between the peptide hydrophobicity and retention times was assessed using SSRCalc (version 3.0) [3] in-built in Skyline (Fig. 2).

**Table 1:** The list of precursor and product ions, their m/z values, charge and collision energies for the optimized assays. Spectral library and individual heavy isotope labelled peptide injections were carried out to choose optimally performing precursors and product ions for each target.

**Gene names**	**Targets**	**Precursor*****m/z***	**Precursor Charge**	**Product*****m/z***	**Product Charge**	**Fragment Ion**	**Collision energy**
EGFR	Y-998	548.9	3	881.3	1	y6	17.2
		548.9	3	679.3	1	y4	17.2
		548.9	3	565.2	1	y3	17.2
		548.9	3	382.2	1	b3 **(quantifier)**	17.2
		548.9	3	566.3	1	b5	17.2
		552.2	3	891.3	1	y6	17.2
		552.2	3	689.3	1	y4	17.2
		552.2	3	575.2	1	y3	17.2
		552.2	3	382.2	1	b3	17.2
		552.2	3	566.3	1	b5	17.2
	Y-1172	772.7	3	538.3	1	y4	26.1
		772.7	3	391.2	1	y3	26.1
		772.7	3	410.2	1	b4	26.1
		772.7	3	523.3	1	b5	26.1
		772.7	3	952.4	1	b9 **(quantifier)**	26.1
		775.3	3	546.3	1	y4	26.1
		775.3	3	399.2	1	y3	26.1
		775.3	3	410.2	1	b4	26.1
		775.3	3	523.3	1	b5	26.1
		775.3	3	952.4	1	b9	26.1
	Y-1197	645.8	2	845.4	1	y6 **(quantifier)**	20.3
		645.8	2	731.3	1	y5	20.3
		645.8	2	660.3	1	y4	20.3
		645.8	2	531.2	1	y3	20.3
		650.8	2	855.4	1	y6	20.3
		650.8	2	741.3	1	y5	20.3
		650.8	2	670.3	1	y4	20.3
		650.8	2	541.2	1	y3	20.3
							
STAT5A	Y-694	433.2	3	712.5	1	y6	12.5
		433.2	3	613.4	1	y5	12.5
		433.2	3	485.3	1	y4 **(quantifier)**	12.5
		435.9	3	720.5	1	y6	12.5
		435.9	3	621.4	1	y5	12.5
		435.9	3	493.3	1	y4	12.5
							
CAV1	Y-14	576.9	3	1078.5	1	y8	18.3
		576.9	3	941.5	1	y7 **(quantifier)**	18.3
		576.9	3	828.4	1	y6	18.3
		576.9	3	385.3	1	y3	18.3
		580.3	3	1088.6	1	y8	18.3
		580.3	3	951.5	1	y7	18.3
		580.3	3	838.4	1	y6	18.3
		580.3	3	395.3	1	y3	18.3
							
DAPP1	Y-139	505.9	3	733.3	1	y5	15.4
		505.9	3	670.3	1	b6 **(quantifier)**	15.4
		509.2	3	743.3	1	y5	15.4
		509.2	3	670.3	1	b6	15.4
							
AHNAK	Y-160	473.2	3	547.3	1	y5 **(quantifier)**	14.1
		473.2	3	671.3	1	b5	14.1
		473.2	3	772.3	1	b6	14.1
		476.6	3	557.3	1	y5	14.1
		476.6	3	671.3	1	b5	14.1
		476.6	3	772.3	1	b6	14.1
	Y-715	449.5	3	772.3	1	b6 **(quantifier)**	13.2
		449.5	3	903.3	1	b7	13.2
		452.2	3	772.3	1	b6	13.2
		452.2	3	903.3	1	b7	13.2
							
NEDD9	Y-164	503.5	3	522.3	1	y4	15.3
		503.5	3	359.2	1	y3 **(quantifier)**	15.3
		503.5	3	615.3	1	b6	15.3
		503.5	3	987.4	1	b8	15.3
		506.9	3	532.3	1	y4	15.3
		506.9	3	369.2	1	y3	15.3
		506.9	3	615.3	1	b6	15.3
		506.9	3	987.4	1	b8	15.3
							
NF1	Y-2579	569.9	3	664.3	1	y5	18
		569.9	3	404.2	1	y3 **(quantifier)**	18
		573.2	3	674.3	1	y5	18
		573.2	3	414.2	1	y3	18
							
INPPL1	Y-1135	763.8	2	1096.5	1	y10	25
		763.8	2	997.4	1	y9	25
		763.8	2	882.4	1	y8	25
		763.8	2	568.3	1	y6 **(quantifier)**	25
		763.8	2	530.3	1	b5	25
		768.9	2	1106.5	1	y10	25
		768.9	2	1007.4	1	y9	25
		768.9	2	892.4	1	y8	25
		768.9	2	578.3	1	y6	25
		768.9	2	530.3	1	b5	25

### 2.2 Immunoaffinity enrichment, LC MS/MS and data analysis

The enrichment of the endogenous phosphotyrosine peptides in the samples was carried out using PhosphoScan kits (Cell Signaling, Danvers, MA). Two antibody kits PTMScan Phospho-Tyrosine Mouse mAb (P-Tyr-100) (product no. 5636) and PTMScan Phospho-Tyrosine Rabbit mAb (P-Tyr-1000) (product no. 14478) were tested to optimize the phosphotyrosine enrichment. Four immunoprecipitations were carried out using the manufacturer׳s protocol on the trypsin digested control peptides from mouse liver extracts (product no. 12219, Cell Signaling, Danvers, MA) and 8 mg of digested protein extract from the H1975 cells using P-Tyr-100 and P-Tyr-1000 kits. The phosphorylated peptides eluted from the antibodies were analyzed on a LTQ-Orbitrap Elite (Thermo Scientific Corp., San Jose, CA) mass spectrometer coupled to a Dionex nLC system (Thermo Scientific Corp., San Jose, CA). Peptides were trapped on a 100 µm i.d. × 2 cm long precolumn (Acclaim PepMap100 Nano Trap column, C18, 5 µm, 100 Å). Subsequent peptide separation was carried out on a nano-LC column (Acclaim PepMap100, C18, 3 µm, 100 Å, 75 µm i.d. × 25 cm, nanoViper). Mobile phase A consisted of 0.1% formic acid in water (v/v) and mobile phase B consisted of 0.1% formic acid in 90% acetonitrile. For each liquid chromatography-tandem mass spectrometry (LC-MS/MS) analysis, peptides were eluted from the column at 300 nL/min using an acetonitrile gradient of 4% B in 5 min, 4–25% B in 70 min, 25–35% B in 85 min, 35–45% B in 95 min and 45–90% B for 10 min. The eluting peptides were interrogated with an Orbitrap analyzer with full scan spectra acquired between *m/z* 350 and *m/z* 1800 at a resolution of 120,000 followed by data-dependent HCD FTMS2 acquisition for the top 15 most abundant ions at 35% normalized collision energy using resolution of 15,000.

**Fig. 3:**
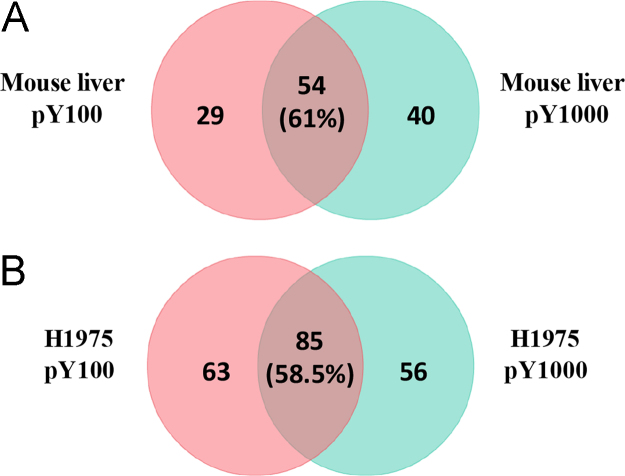
Venn diagrams showing the number of unique and commonly identified phosphotyrosine sites from P-Tyr-100 and P-Tyr-1000 immunoprecipitations in A) control digested peptides from mouse liver extracts and B) digested protein extracts from H1975 cells.

Raw MS files were searched against the Uniprot human and mouse proteome database using the Maxquant software (version 1.5.7.4) with Andromeda search engine. Search parameters included cysteine carbamidomethylation as a fixed modification and phosphorylation (STY) was added as a variable modification. The digestion mode was set to specific with trypsin as the digestion enzyme and two missed cleavages were allowed. Mass tolerances were set to 6 ppm for precursor ions and 20 ppm for product ions. The search criteria further included false discovery rates of 0.01 for both protein and peptide identifications. The minimum peptide length was 7 amino acid residues. Decoy database search was activated and the database searching was supplemented with the common contaminants often found in cell culture and proteomics sample preparation experiments; these were later identified and removed. All the other settings were set to default except that the “match between runs” feature was enabled with the default settings. The identification data for the phoshopeptides from these experiments is shown in Supplementary table 1. There was only 60% overlap (common and unique ids listed in Supplementary table 1) in the phosphopeptides identified from the two kits (Fig. 3A, B). Hence, we used a combination of antibodies to enrich our samples. The final optimized enrichment protocol comprised a combination of P-Tyr-100 and P-Tyr-1000 antibody slurries at 1:1 v/v.

### 2.3 Estimation of dwell times, quantitative data and analysis of the replicates

**Fig. 4:**
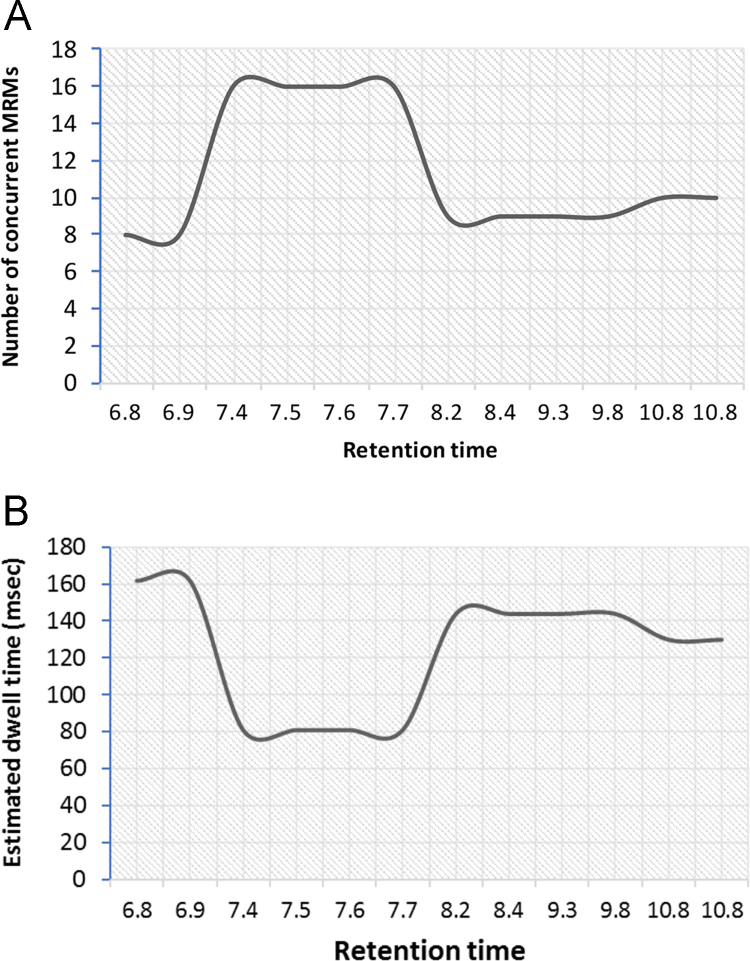
Graphs showing A) number of concurrent transitions being measured and B) estimated dwell time across the chromatographic elution of the targets for the final optimized scheduled MRM assays using a 2-min retention time window.

**Fig. 5:**
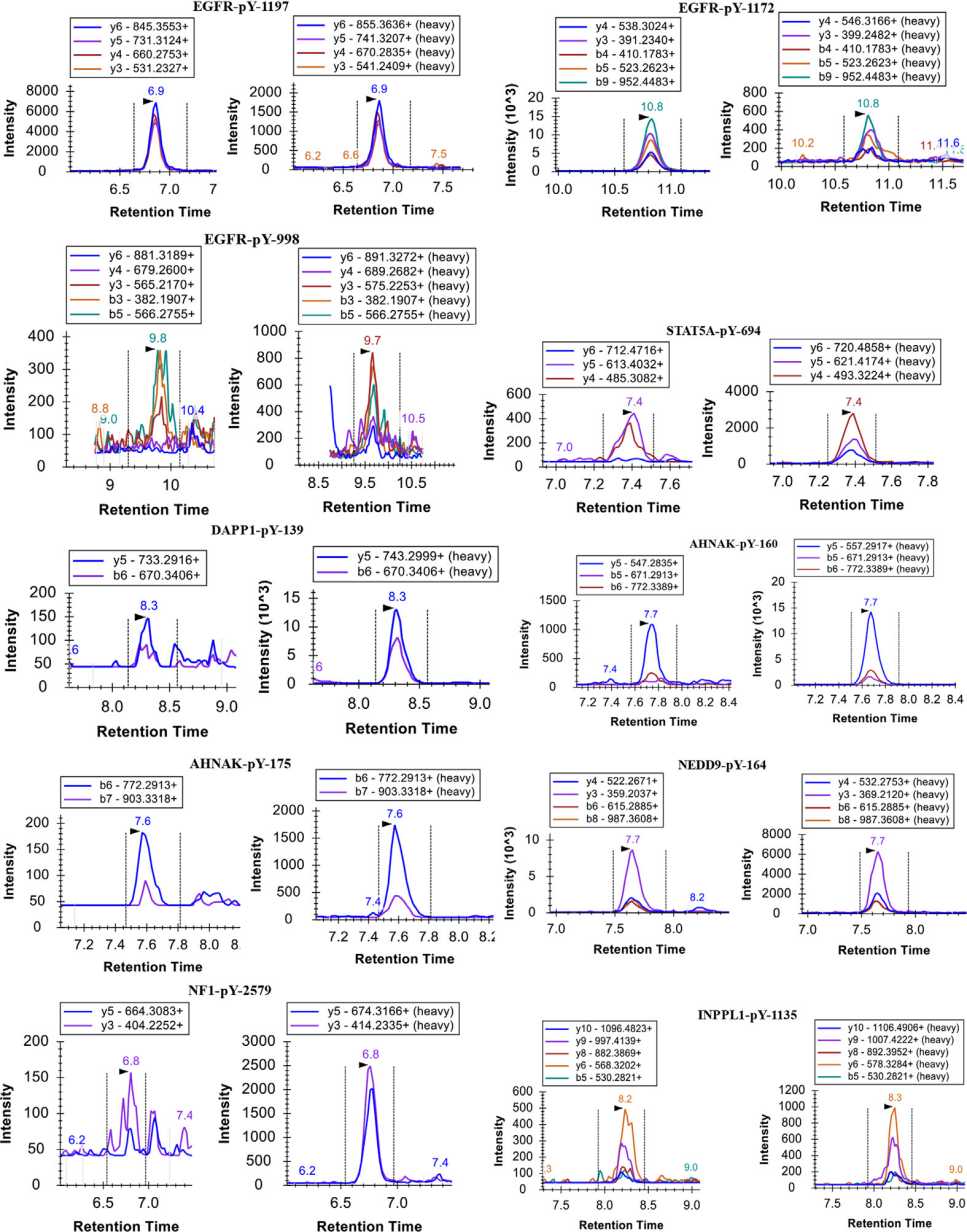
Representative chromatographic profile for the endogenous (left) and the heavy labelled internal standards (right) for the H1975 (DMSO/vehicle treated) cells.

**Fig. 6:**
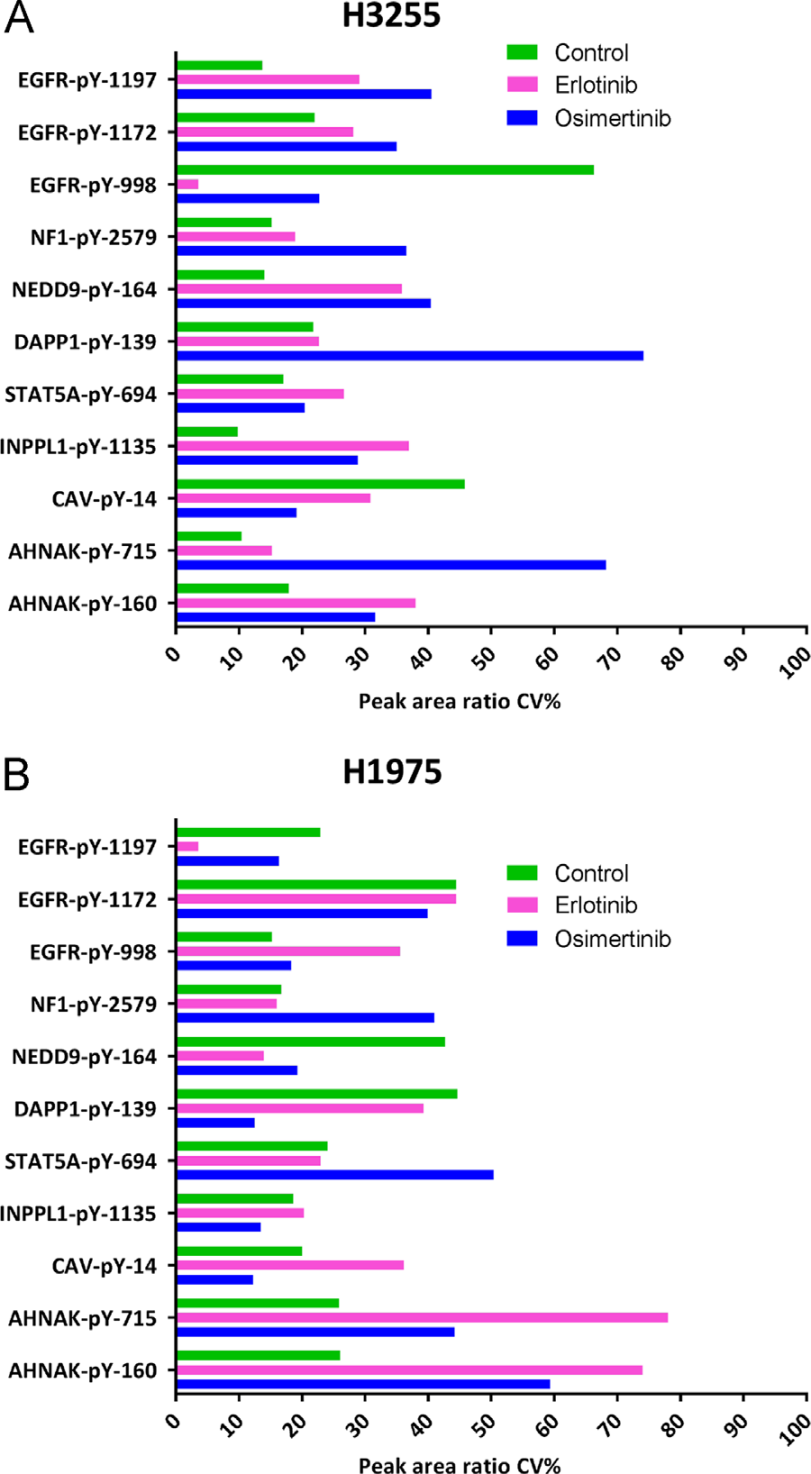
Peak area ratio coefficient of variations obtained from three biological replicates for the relative quantification in A) H3255 and B) H1975 cells for DMSO/vehicle, erlotinib and osimertinib treatments.

The final chromatographic scheduled methods consisted of a 25-minute gradient with 2-minute retention time windows. The number of concurrent transitions being measured in any retention time window varied from 8 to 16 (Fig. 4A). As the target peptides eluted, at any given time in the gradient, the dwell times were estimated to fall in the range of 80–160 milliseconds (Fig. 4B). This allowed for excellent sensitivity as we acquired around 20 points across the chromatographic peak for all the “quantifier” transitions. The chromatographic profiles obtained from these optimized methods in H1975 cells is shown in Fig. 5. The quantitative data associated with all experiments (control and TKI treatments) has been summarized in Supplementary Table 1, [1]. The CVs for the peak area ratios obtained from implementing these assays in H3255 and H1975 lung adenocarcinoma cells with and without erlotinib and osimertinib treatment are shown (Figs. 6A and 6B).

### 2.4 Quantitative assay characterization and calibration curve generation

**Fig. 7:**
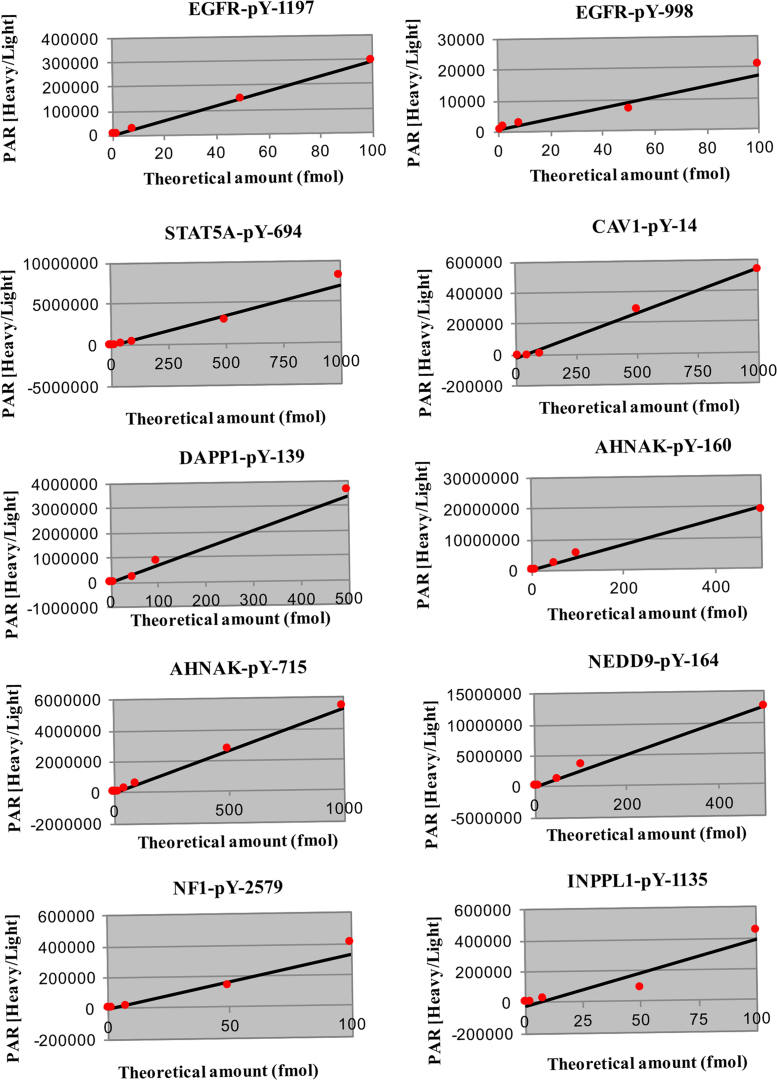
Response curves for the phosphotyrosine targets for quantitative analysis. Linear regression was used to fit the data points using a 1/y weighting for each concentration.

Quantitation was carried out using synthetic peptide standards which were synthesized as matched pairs of light and heavy stable isotope-labeled peptides (New England Peptide, Gardner, MA). Heavy peptides were ^13^C and ^15^N labelled at the C-terminal lysine or arginine position of the tryptic peptide target. A reverse response curve was generated in digested and phosphotyrosine enriched matrix of H1975 cells treated with DMSO and processed in a similar manner to the TKI treated samples. For the calibration samples, the light peptide amount was held constant (2 fmol) and the heavy peptide was varied over a range (0.01, 0.1, 0.5, 2, 8, 50, 100, 500, 100 fmol). The analytical performance of the quantitative assays was characterized by determining the linear dynamic range and figures of merit like limit of detection (LOD) and lower limit of quantification (LOQ) before their application in the lung adenocarcinoma cells as described in [1]. The calibration curves are shown in Fig. 7.

### 2.5 cBioPortal analysis of the target genes

**Fig. 8:**
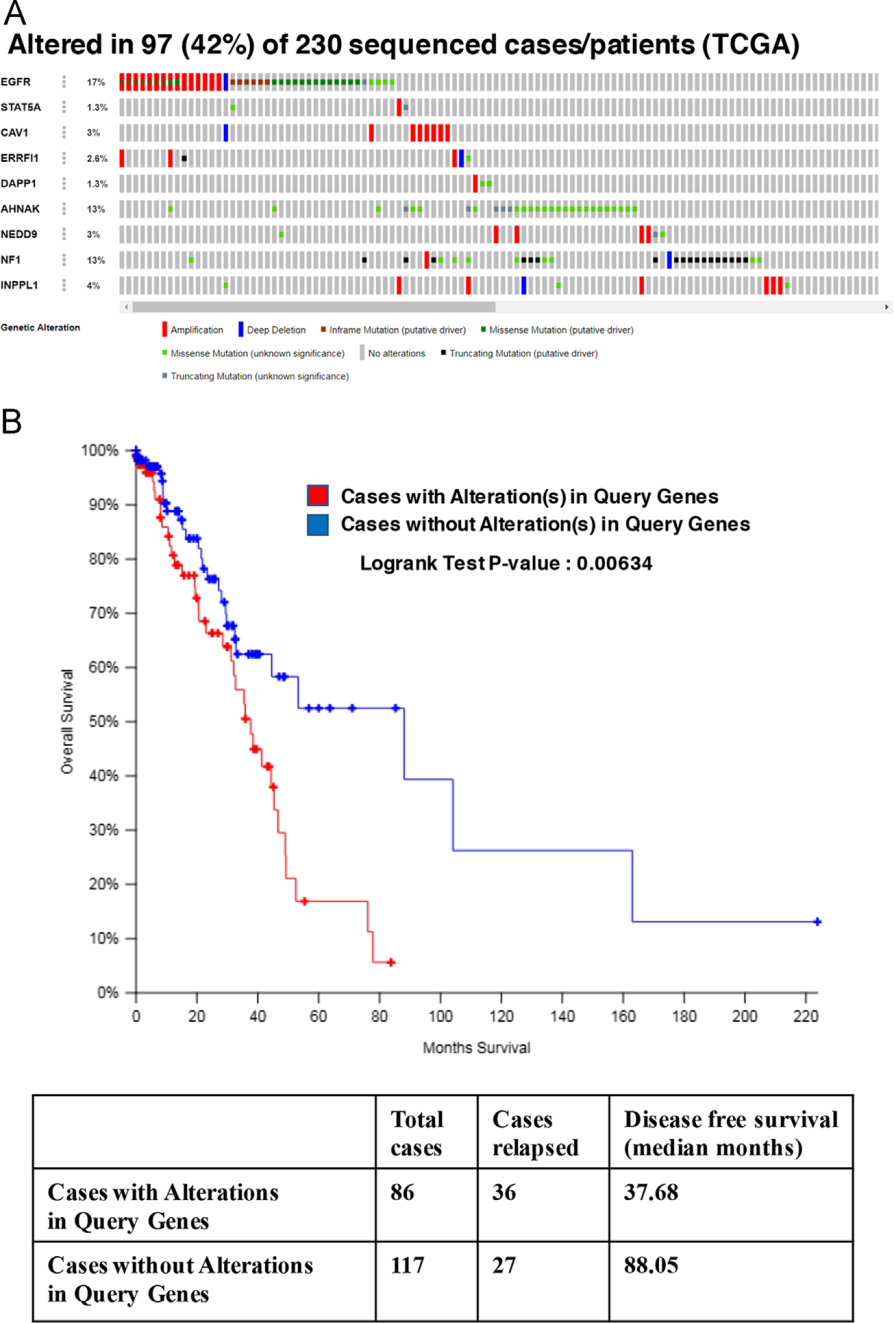
cBioPortal query of the TCGA lung adenocarcinoma dataset (4) A) for alterations in the target list and B) correlation with disease-free survival.

**Fig. 9:**
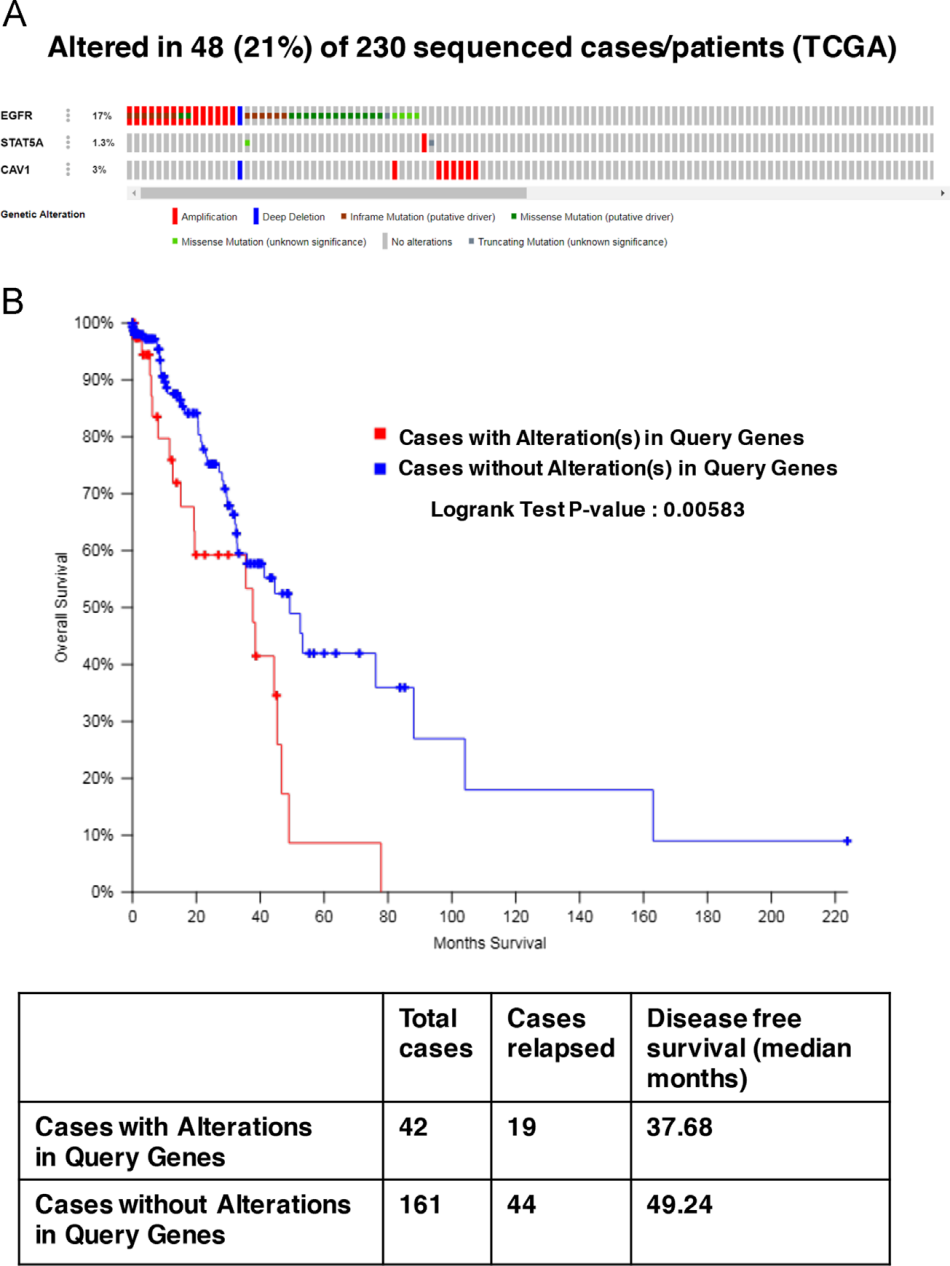
cBioPortal query of the TCGA lung adenocarcinoma dataset (4) A) for alterations in targets EGFR, CAV1 and STAT5A and B) correlation with disease-free survival.

The target list from this study was queried against the TCGA lung adenocarcinoma dataset [4] through cBioPortal [5], [6] for alterations including missense, truncating, in-frame mutations, amplification, deletions, mRNA up- and downregulation and protein up- and down regulation by RPPA assay. The results showed that the target list was altered in 42% of the 230 sequenced patients (Fig. 8A) and the disease-free survival among patients with alterations in the target genes was significantly lower (Logrank test P-value:0.00634) (Fig. 8B). The query against the same patient database for the targets EGFR, CAV1 and STAT5A identified alterations in 21% of the 230 sequenced patients (Fig. 9A) and a significantly lower disease-free survival (Logrank test P-value:0.00583) (Fig. 9B).

## Appendix A Supplementary material

**Supplementary Table 1:** Phosphopeptide identification data from the P-Tyr-100 and P-Tyr-1000 enrichment kits for the digested mouse liver extract (control) and H1975 extracts. The common and unique phosphotyrosine sites obtained from the two enrichment kits are also listed.Supplementary material
