# Supplementary material for: Dataset describing the development, optimization and application of SRM/MRM based targeted proteomics strategy for quantification of potential biomarkers of EGFR TKI sensitivity
Source: Data Brief. 2018 May 2;19:424–36. doi: 10.1016/j.dib.2018.04.086 (PMC5997585; doi:10.1016/j.dib.2018.04.086)
Supplement: Supplementary file 1 — Supplementary material [file mmc1.pdf]

Conflict of Interest:

The authors declare no conflicts of interest.
